# Automated LVO detection and collateral scoring on CTA using a 3D self-configuring object detection network: a multi-center study

**DOI:** 10.1038/s41598-023-33723-w

**Published:** 2023-05-31

**Authors:** Omer Bagcilar, Deniz Alis, Ceren Alis, Mustafa Ege Seker, Mert Yergin, Ahmet Ustundag, Emil Hikmet, Alperen Tezcan, Gokhan Polat, Ahmet Tugrul Akkus, Fatih Alper, Murat Velioglu, Omer Yildiz, Hakan Hatem Selcuk, Ilkay Oksuz, Osman Kizilkilic, Ercan Karaarslan

**Affiliations:** 1 Radiology Department, Sisli Hamidiye Etfal Research and Training Hospital, Istanbul, Turkey; 2 Radiology Department, School of Medicine, Acibadem Mehmet Ali Aydinlar University, Istanbul, Turkey; 3 Neurology Department, Istanbul Istinye State Hospital, Istanbul, Turkey; 4 School of Medicine, Acibadem Mehmet Ali Aydinlar University, Istanbul, Turkey; 5 Artificial Intelligence, and Information Technologies, Hevi AI Health, Istanbul, Turkey; 6 Radiology Department, Cerrahpaşa Medical Faculty, Istanbul University-Cerrahpasa, Istanbul, Turkey; 7 Radiology Department, School of Medicine, Erzurum Ataturk University, Istanbul, Turkey; 8 Radiology Department, Istanbul Fatih Sultan Mehmet Training and Research Hospital, Istanbul, Turkey; 9 Radiology Department, Istanbul Bakırköy Sadi Konuk Training and Research Hospital, Istanbul, Turkey; 10 Computer Engineering Department, Istanbul Technical University, Istanbul, Turkey

**Keywords:** Cerebrovascular disorders, Software

## Abstract

The use of deep learning (DL) techniques for automated diagnosis of large vessel occlusion (LVO) and collateral scoring on computed tomography angiography (CTA) is gaining attention. In this study, a state-of-the-art self-configuring object detection network called nnDetection was used to detect LVO and assess collateralization on CTA scans using a multi-task 3D object detection approach. The model was trained on single-phase CTA scans of 2425 patients at five centers, and its performance was evaluated on an external test set of 345 patients from another center. Ground-truth labels for the presence of LVO and collateral scores were provided by three radiologists. The nnDetection model achieved a diagnostic accuracy of 98.26% (95% CI 96.25–99.36%) in identifying LVO, correctly classifying 339 out of 345 CTA scans in the external test set. The DL-based collateral scores had a kappa of 0.80, indicating good agreement with the consensus of the radiologists. These results demonstrate that the self-configuring 3D nnDetection model can accurately detect LVO on single-phase CTA scans and provide semi-quantitative collateral scores, offering a comprehensive approach for automated stroke diagnostics in patients with LVO.

## Introduction

According to the World Health Organization, 15 million people suffer from a stroke attack every year, resulting in 6 million deaths and 5 million disabilities^1^. Ischemic stroke with large vessel occlusion (LVO) is the most severe type of ischemic stroke, and approximately one-third of ischemic strokes are caused by LVO^2^. Mechanical thrombectomy is the standard treatment for acute ischemic stroke with LVO, as several large-scale studies in 2015 demonstrated its benefits in restoring blood flow in eligible patients^3,4^.

Early diagnosis of LVO is crucial for the success of mechanical thrombectomy, as the chances of achieving functional independence decrease with delays^5^. Computed tomography angiography (CTA) has become the standard for acute stroke imaging in assessing LVO^6^. It also provides information about collateral status, which is useful for decision-making in mechanical thrombectomy^4,7,8^. The severe consequences of delayed diagnosis of LVO and the inter-observer variations among radiologists in assessing collateral status in ischemic stroke patients necessitates automated diagnosis^9,10^.

There has been a significant amount of research on the automated detection of LVO and collateral scoring on CTA. Some earlier studies used traditional image processing methods or machine learning to assess stroke patients with LVO, but the performance and generalizability of these methods were poor^11,12^. Several recent studies have used deep learning (DL) to identify LVO or score collateral status on CTA with promising results. However, most earlier studies were limited by the use of single-center data^13,14^, lack of independent external test sets^13–15^, use of classification models providing binary outputs without locating or segmenting LVO^16,17^, use of data from similar patient demographics^14,15,17,18^ (i.e., primarily clinical trial data obtained in high-income countries), and use of commercial solutions without a detailed description of the underlying DL methods^10,17,18^.

In this study, we approached the detection of LVO and collateral scoring on CTA as a multi-task 3D object detection problem using a state-of-the-art self-configuring nnDetection model on a large-scale multi-center and multi-vendor data set derived from an emerging country. We used this model to identify LVO and assess the collateral status on single-phase CTA, then assess its performance on external data.

## Methods

Acibadem Mehmet Ali Aydinlar University review board approved this retrospective multi-center study and waived the need for informed consent. Radiologists from six tertiary centers retrospectively searched their hospital and radiology information systems using several keywords for adult patients who underwent CTA referred from the emergency services between January 2018 and June 2022 for the suspicion of LVO.

We included patients with accompanying pathologies (e.g., hemorrhage, metal artifacts, mild motion) to test the real-world performance and applicability of the model. In addition, we included scans with different contrast phases (early arterial, peak arterial, arterio-venous, early venous, late venous) for the same purpose. We excluded scans with severe motion artifacts and without contrast opacification as determined by the radiologists.

**Figure 1:**
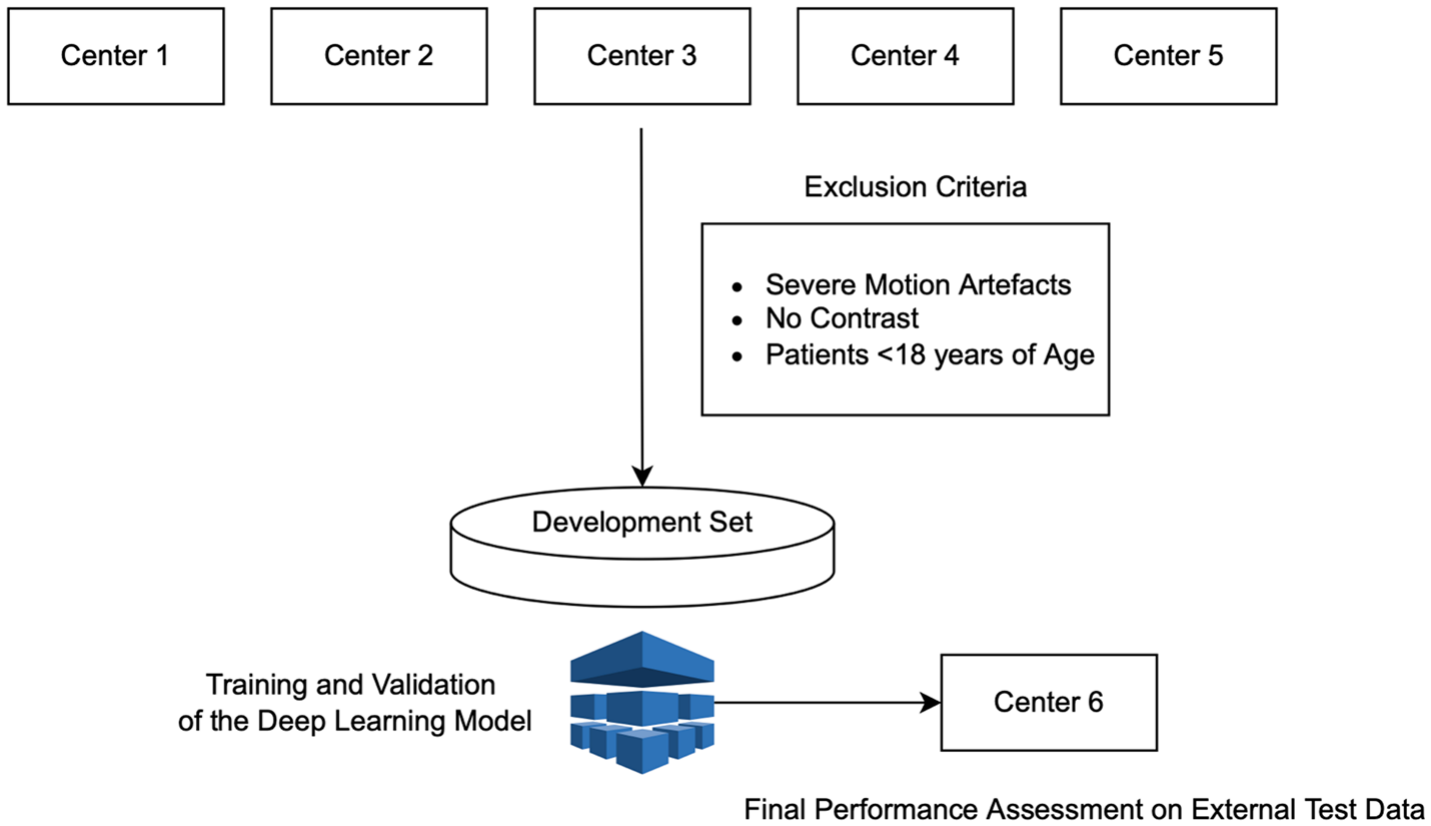
The study sample selection along with the model training and selection processes. We collected brain computed tomography angiography scans from Centers 1–5 and excluded patients following the exclusion criteria. The development set had 2425 patients with 435 large vessel occlusion positive scans. We train our nnDetection model on the development set and tested the model’s performance on the test data from Center 6.

We divided the dataset into a development sample for training and validating the DL models and an external test sample to test the unbiased performance of the DL models in identifying LVO and assigning collateral scores. We used CTA scans from 5 out of 6 centers for the development set and the remaining center's CTA scans for the test set. The CTA scans in the development set were obtained with scanners from Siemens (n = 6), GE Healthcare (n = 4), Philips (n = 1), and Canon (n = 1). The minimum number of detector rows was 32 for the development set, and the maximum slice thickness was 1.5 mm. The test set was obtained using Siemens (n = 1) and Canon (n = 1) scanners. The minimum number of detector rows was 64, and the maximum slice thickness was 1 mm. Figure 1 shows the patient enrollment process of the study.

### Ground-truth annotations

Two radiologists (D.A. and O.B.) with 6 and 4 years of stroke imaging experience independently assessed the scans for the presence of LVO. All scans were analyzed using a dedicated online platform (MD.ai, https://docs.md.ai). First, all raw CTA images were pre-processed and saved as thick-slab 20-mm maximum-intensity-projection (MIP) images at 5 mm overlapping intervals using the PIL library of the Python programming language. We chose to use MIP images instead of raw CTA images because they enable human readers to more effectively identify the presence of LVO^19^. Moreover, we believe that employing MIP images instead of raw CTA images may also enhance the learning process of the model, as deep learning models are trained and optimized based on human visual outputs (i.e., ground-truth labels).

LVO was considered as the occlusion of the terminal internal carotid artery (ICA-T), the M1 segment of the middle cerebral artery (MCA-M1), or the proximal part of the M2 segment of the MCA (MCA-M2). The M1 segment was defined as the MCA from the ICA-T to the MCA bifurcation or trifurcation; the proximal M2 segment was defined as the MCA from the bifurcation/trifurcation up to the midpoint of the Sylvian fissure on the coronal plane^20^. We did not consider patients with occlusion in the anterior or posterior cerebral arteries as LVO cases.

The single-phase CTA collaterals in the occluded MCA territory were graded as proposed by Tan et al.^21^ as follows: no collateral filling (Grade 0); ≤ 50% collateral filling (Grade 1); > 50% but < 100% collateral filling (Grade 2); and 100% collateral filling (Grade 3). The scans were dichotomized as having poor collaterals for Grades 0 and 1 and good collaterals for Grades 2 and 3 following earlier studies^22^.

The discrepancies were resolved by a neuroradiologist (O.K.) with 20 years of diagnostic and interventional neuroradiology experience in a joint meeting, and each scan's final LVO and collateral status were assigned. After the joint meeting and determination of the scan-level final ground truth LVO and collateral scores, one of the radiologists (O.B. or D.A.) placed 3D-boxes onto the LVO (LVO-positive box) covering the virtual trajectory of ICA-T, MCA-M1, and MCA-M2 and the contralateral side covering normal ICA-T, MCA-1, and MCA-M2 segments (LVO-negative box). Furthermore, the same radiologists placed 3D boxes for collaterals along with their collateral scores (good or poor) onto two brain hemispheres, starting from the basal ganglia level and ending at the top of the lateral ventricles.

### Deep learning model

**Figure 2:**
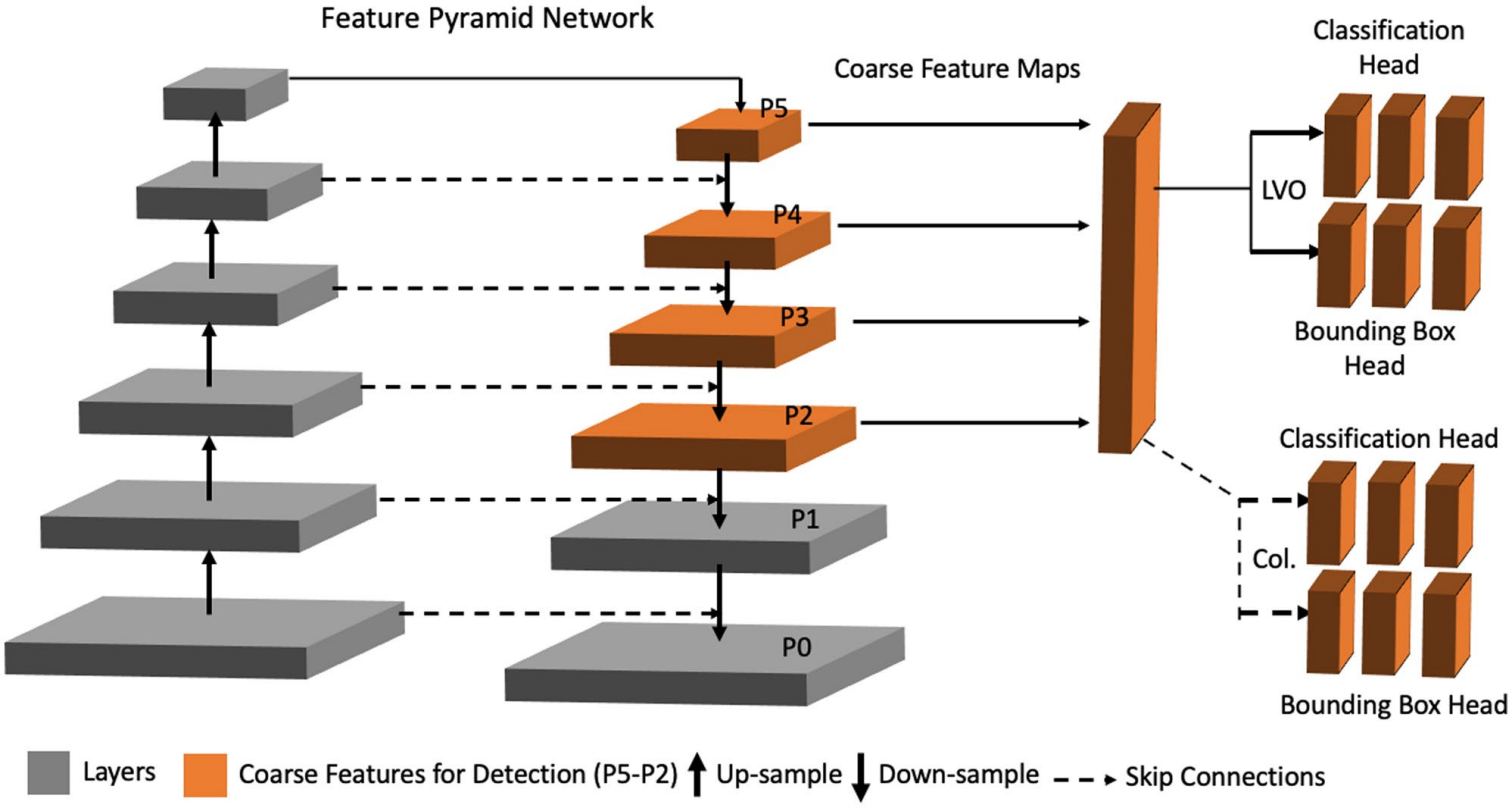
The representative diagram of the nnDetection Model. The backbone of nnDetection is a pyramid-like network with bottom-up (left) and top-down (right) pathways with interconnected layers. The upper layers have a lower spatial resolution yet have representative features for the task at hand. The classification and regression (i.e., bounding boxes) were carried out on the representative averaged feature maps. On the bottom-up path the spatial resolution of the feature maps decreases while getting richer and denser information, while the top-down recovers the spatial dimension. The skip connection between the pathways facilitates information flow. The orange-colored features maps (P5–P2) are used for the object detection and classification. In this study, there was a primary task used in every scan: (1) the identification of the zone of the middle cerebral artery (regression) and the large vessel occlusion (classification); and one auxiliary task used in patients with large vessel occlusion: the branch for the identification of the brain hemisphere (regression) where the collateral scoring will be made by the classification branch.

We used state-of-the-art 3D self-configuring medical object detection DL method, nnDetection method, for the present study^23^. The nnDetection selects the best architecture depending on the dataset by adhering to a set of inter-dependent principles: (1) data fingerprint, which covers the relevant properties of the training data; (2) rule-based parameter, which employs a set of heuristics based on the fingerprint; (3) fixed parameters, which do not rely on the data; and (4) empirical parameter optimization, which is the set of parameters optimized during the training. The nnDetection was built on the top of the Retina U-net, using a similar network topology^24^. Figure 2 demonstrates the schematic representation of the baseline topology used in the present study.

The input of nnDetection was the stack of 3D-MIP slices of a CTA scan, and the model output was the ground-truth 3D bounding boxes with their corresponding categories (i.e., LVO-positive and LVO-negative 3D boxes and the 3D collateral boxes). We used stratified fivefold cross-validation during the training. Briefly, the development set was partitioned into five folds, with four of the folds used to train the model, while the remaining fold served as the validation data to select the best-performing model through the epochs. We used a stratified split ensuring that the distribution of the target classes is preserved in each fold. After the training, five different models were obtained, and the ensemble of these five models was used to predict the presence of LVO and collateral scoring on the external test data.

The sum of cross-entropy (classification) and generalized intersection-over union (regression) loss was used for the training. The model was trained for 60 epochs. The batch size was set at 4, and half of the batch was forced to include at least one ground-truth box. The model was optimized with stochastic gradient descent with a Nesterov momentum of 0.9, beginning the training with a linearly ramped learning rate from 1e−6 to 1e−2 over the first 4000 iterations. After that, the poly learning rate schedule was used until epoch 50. The last 10 epochs were trained with a cyclic learning rate fluctuating between 1e−3 and 1e−6 during every epoch. The standard data augmentation scheme of nnU-Net, which includes Gaussian noise, Gaussian blur, brightness, contrast, simulation of low resolution, gamma augmentation, elastic deformation, scaling, flipping, mirroring, and rotation, was implemented on-the-fly during the training process.

### Statistical analyses

Statistical analysis was performed using Scipy library v1.5.4 of Python programming language ("https://docs.scipy.org"). The pair-wise inter-rater agreements were assessed using Cohen's kappa^25^. The kappa scores were interpreted as follows: a kappa score of < 20, a poor agreement; 21–40, a fair agreement; 41–60, a moderate agreement; 61–80, a good agreement; and 81–100, an excellent agreement. All performance metrics for the DL models were calculated and presented on a scan basis. The model’s predictions were binarized using the probability threshold of ≥ 0.6 for calculating diagnostic metrics. The primary metric for investigating a model's performance was diagnostic accuracy accepting the ground-truth annotations as the reference. Other metrics used for assessing models' performance were sensitivity, specificity, negative predictive value (NPV), positive predictive value (PPV), and F1-score. The 95% confidence intervals were exact for F1-score, accuracy, sensitivity, and specificity, while were the standard logit confidence intervals for negative predictive value and positive predictive value^26,27^.

### Ethical approval and consent to participate

All procedures were performed in studies involving human participants following the ethical standards of the institutional and/or national research committee and with the 1964 Helsinki declaration.

## Results

A total of 2425 patients, 1746 men (72%), with a mean age of 55.74 ± 14.34 years (range 30–90), were enrolled in the development sample. There were 435 LVO-positive patients (17.79%) in the development sample. The consensus of radiologists assigned the status of collaterals as good in 200 and poor in 235 patients in the development set.

The external test data consisted of 345 CTA patients with a mean age of 58.34 ± 12.34 years (range 40–86). In the test data, 81 out of 345 scans (23.47%) were annotated as LVO-positive by the consensus of radiologists.

The consensus of radiologists assigned the status of collaterals as good in 40 out of 81 (49.40%) and poor in 41 (50.60%) in the external test set. There was a moderate agreement between radiologists with a kappa score of 0.62 in the initial independent readings in assigning collateral scores. The first and second radiologists had good and moderate agreement with a kappa score of 0.82 and 0.75 compared with the consensus collateral scores. Notably, DL-based collateral scores had a kappa of 0.80, equating to a good agreement with the consensus readers.

The nnDetection model yielded a diagnostic accuracy of 98.26% (95% CI 96.25–99.36%) in identifying LVO by correctly classifying 339 out of 345 CTA scans in the external test set. The F1-score, sensitivity, specificity, NPV, and PPV of the model on the external test set were 96.30% (95% CI 88.46–99%), 96.30% (95% CI 87.56–99.23%), 98.86% (95% CI 96.72–99.77%), 98.86% (95% CI 96.63–99.62%), and 96.30% (95% CI 89.40–98.77%), respectively.

Of the 345 scans, 165/345 (47.82%) were arterial phase, and 180/345 (52.18%) were venous phase. The model's F1-Score, accuracy, sensitivity, specificity, NPV, and PPV for the arterial phase were 94.24% (95% CI 80.28–100%), 96.97% (95% CI 93.07–99.01%), 93.18% (95% CI 81.34–98.57%), 98.35% (95% CI 94.16–99.8%), 97.54% (95% CI 92.98–99.49%), and 95.35% (95% CI 84.19–99.43%). For the venous phase, they were 98.59% (95% CI 92.10–100%), 99.44% (95% CI 96.94–99.99%), 100% (95% CI 90.51–100%), 99.3% (95% CI 96.17–99.98%), 100% (95% CI 97.44–100%), and 97.37% (95% CI 86.19–99.93%), respectively.

**Figure 3:**
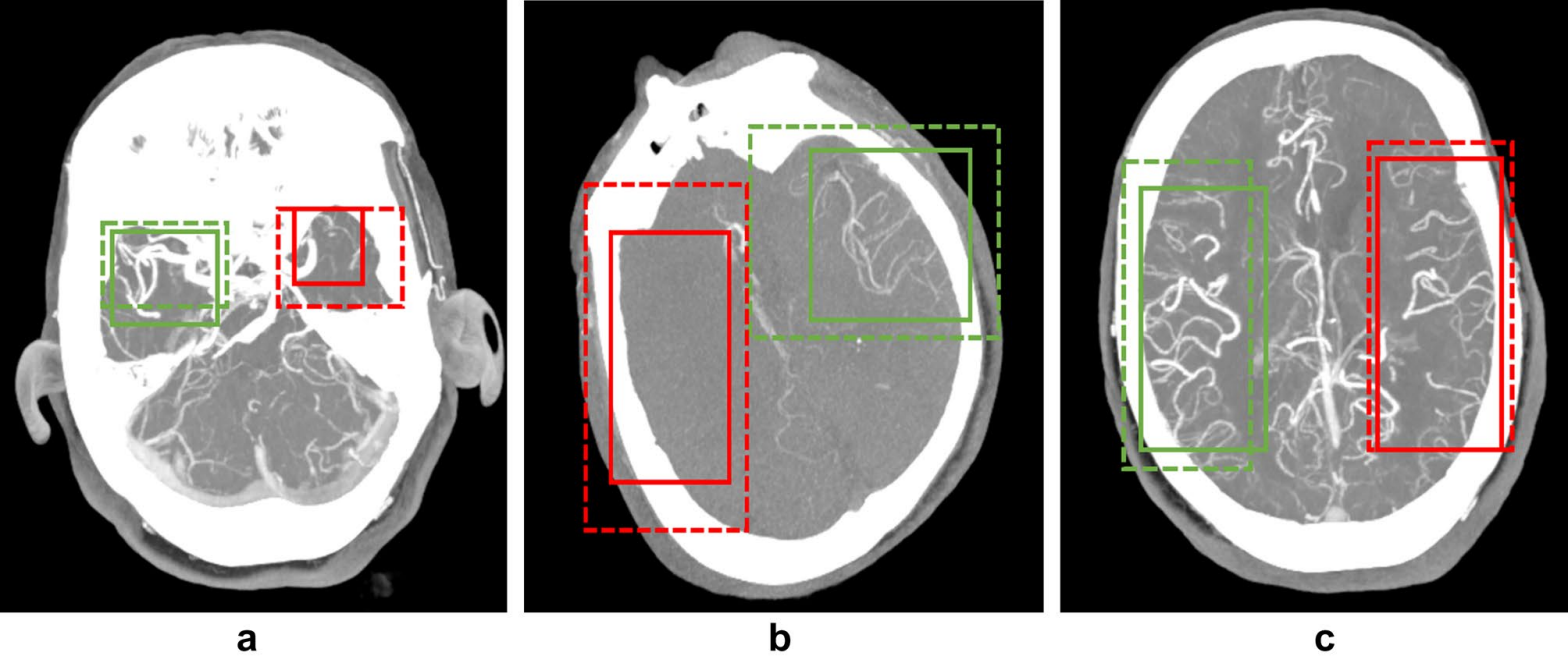
The examples of correct predictions by the deep learning model. The predictions of the model are shown with a dashed box, while the ground-truth box with continuous lines. The red colors indicate the side with pathology while the green colors indicate the normal side. (**a**) The deep learning model correctly identified the left middle cerebral artery M1 segment occlusion. (**b**) The deep learning model correctly assigned collateral status as poor in a patient with right middle cerebral artery M1 segment occlusion. (**c**) The deep learning model correctly assigned collateral status as good in a patient with left middle cerebral artery M1 segment occlusion.

**Figure 4:**
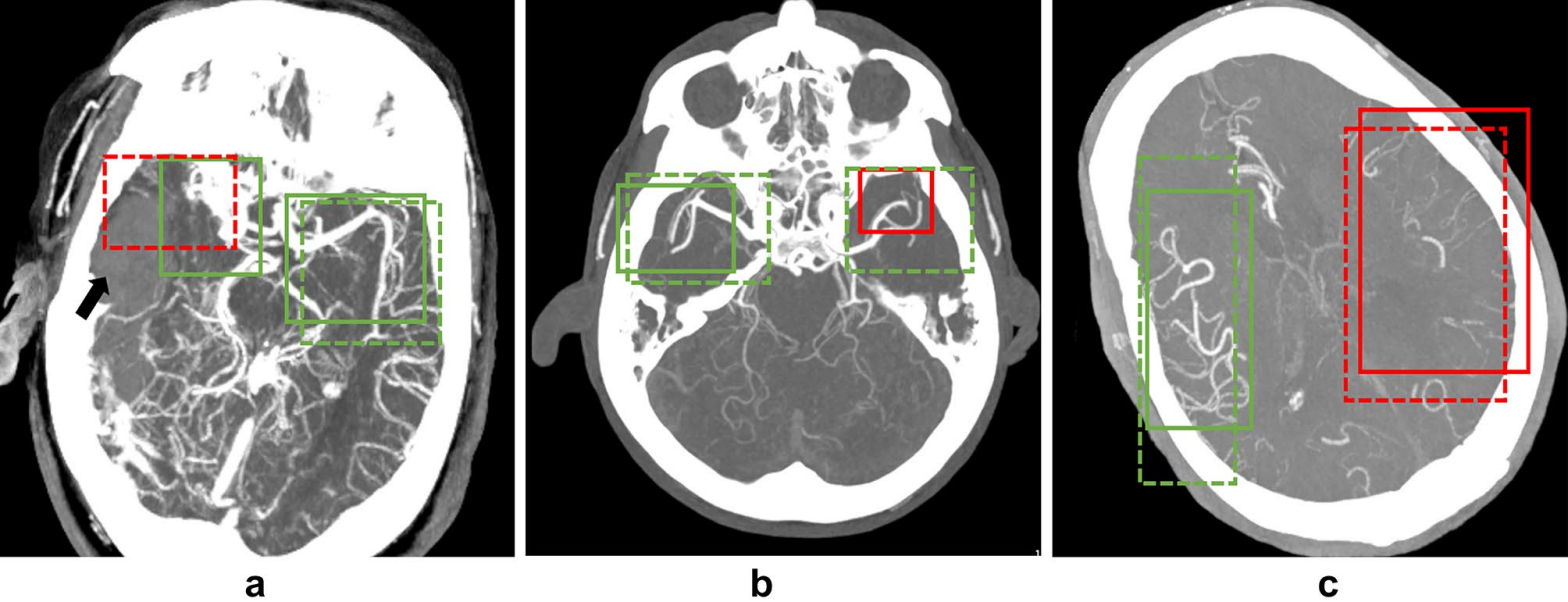
Examples of false predictions by the deep learning model. (**a**) The deep learning model incorrectly identified LVO in the right middle cerebral artery M1 segment. In this patient, a large intraparenchymal hematoma (arrow) displaces the middle cerebral artery medially and upwards. Hence, the model probably failed to identify the normal contrast filling of the artery and made an incorrect prediction. (**b**) The deep learning model failed to identify the left middle cerebral artery proximal M2 segment occlusion. (**c**) The deep learning model incorrectly assigned collateral status as good in a patient with left middle cerebral artery M1 segment occlusion. The experts assigned collateral status as poor in this patient.

**Figure 5:**
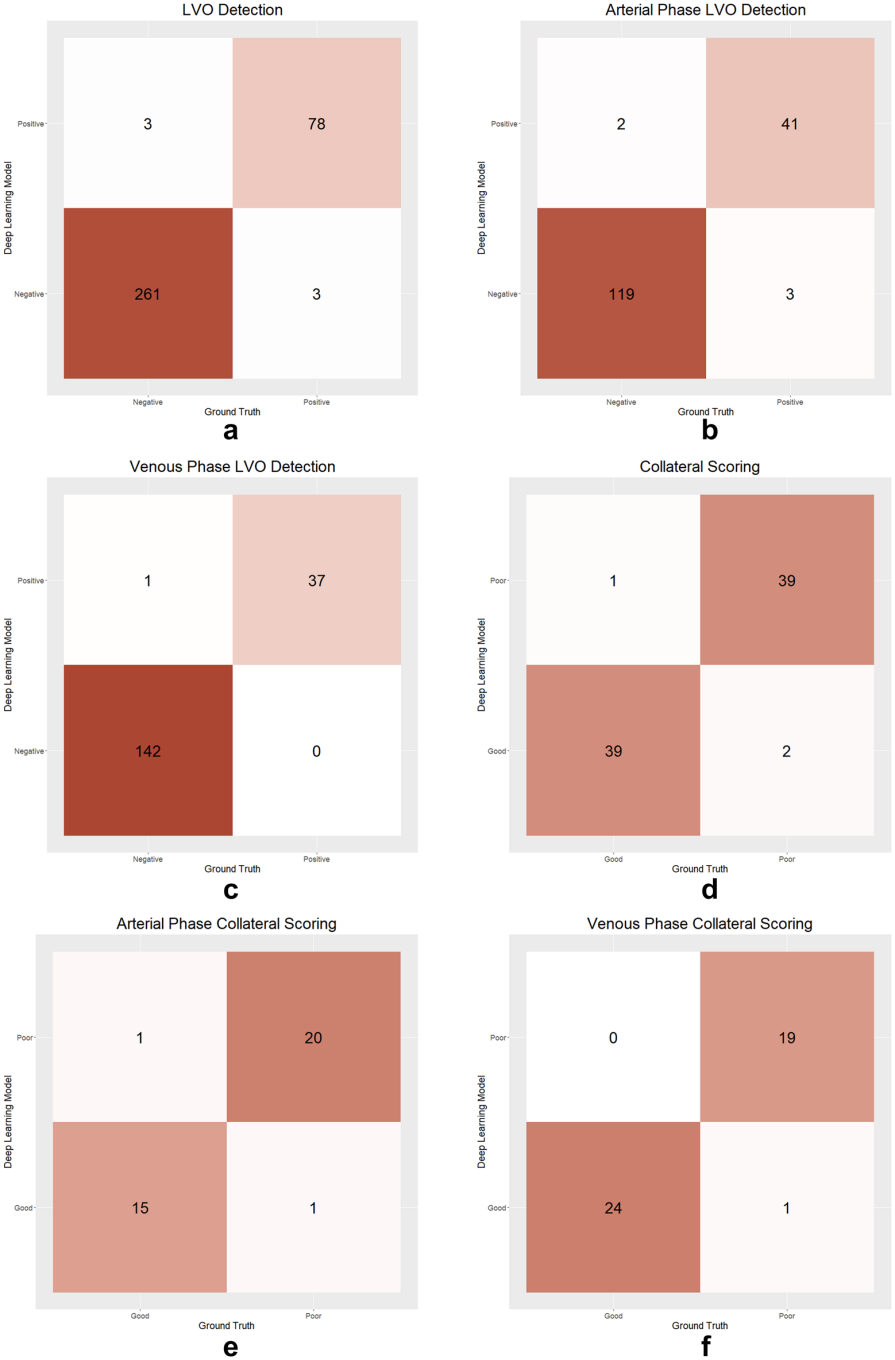
Confusion matrices displaying the deep learning model's predictions compared to the ground-truth labels. The figure illustrates the model's performance in detecting large vessel occlusion on the overall test set (**a**), arterial-phase scans (**b**), and venous-phase scans (**c**). Additionally, the model's performance in collateral scoring is presented for the test set (**d**), arterial-phase scans (**e**), and venous-phase scans (**f**).

Figures 3 and 4 display representative cases illustrating the model's predictions. Figure 5 showcases confusion matrices that compare the model's predictions with ground-truth labels for LVO detection and collateral scoring assessments.

## Discussion

This study addressed the challenge of detecting LVO in CTA scans by treating it as a 3D object detection problem, utilizing the state-of-the-art self-configuring medical object detection method, nnDetection. The model, trained on a large-scale, multi-center, heterogeneous dataset (e.g., various centers, scanner vendors, CTA acquisition protocols, contrast phases, etc.), achieved an accuracy exceeding 98% in identifying LVO on independent external test data. Moreover, the nnDetection model demonstrated strong agreement in assigning collateral scores, exhibiting performance comparable to or surpassing individual radiologists' reliability when considering the radiologists' consensus as ground truth. Importantly, while the nnDetection model displayed marginally higher LVO detection performance in venous-phase CTA, it exhibited exceptional performance in both arterial-phase and venous-phase CTA for LVO detection and collateral scoring, highlighting its robustness and generalizability.

In this work, we opted to use an object detection model instead of a DL model that provides scan-level or slice-level predictions (i.e., LVO-positive slice or scan). This decision was based on two key factors. First, in the context of LVO detection on CTA, bounding boxes (i.e., object detection) supply potent supervision signals for the learning process while reducing the need for resource-intensive pixel-level annotations (i.e., segmentation)^28,29^. Second, presenting the model's predictions for LVO detection enables users to understand how the model reached its decision in a specific case, potentially improving the reliability and explainability of the predictions.

We chose to use nnDetection for our study over potential alternatives such as Retina U-Net, owing to its capabilities in handling multi-class 3D object detection tasks within a unified framework^23^. This framework is specifically designed for medical imaging tasks and provides a flexible platform that allows seamless integration of various architectures, including Retina U-Net. By leveraging nnDetection, we were able to focus on the specific problem of LVO detection while taking advantage of an established and well-supported framework. Specifically designed for medical imaging tasks, nnDetection offers a flexible framework that allows seamless integration of various architectures, including Retina U-Net. This framework also provides automatic data handling, augmentation, and preprocessing, which streamlines the development process and ensures consistency across experiments. Furthermore, nnDetection has demonstrated strong performance in numerous medical imaging challenges, highlighting its effectiveness in addressing a wide range of tasks^23^.

In our study, we observed that most false-negative cases were M2 occlusions, which are inherently difficult to detect, even for less-experienced radiologists. False-positive cases primarily arose due to accompanying pathologies, such as intracerebral hematomas. To address these issues, we propose adding more distal occlusion cases to the training dataset, developing a specialized model for challenging cases, or increasing the number of false-positive inducers in the training data to enhance the model's performance. Additionally, implementing safeguards before or after the deep learning model for LVO detection, such as training a model to identify hematomas and halt the subsequent LVO detection process or alert users to potential risks, could help improve the accuracy of the system.

Sawicki et al. explored the performance of a commercially available DL software (e-Stroke, Brainomix Ltd., Oxford, UK) in assessing LVO in a single-center retrospective study^30^. The authors found that the software provided low performance with a diagnostic accuracy of 77%, which was lower than the DL model in this study by a large margin. Based on their investigations, the authors stated that the software had an acceptable performance for MCA-M1 occlusion while falling short for MCA-M2 occlusion. Strikingly, the software showed a similar diagnostic performance to that of a medical student in their work. Unfortunately, the authors did not provide adequate detail regarding the inner working of the software used in their study.

Grunwald et al. investigated the yields of the same commercial software (e-Stroke, Brainomix Ltd., Oxford, UK) in assigning collateral scores using single-phase CTA^10^. The ground-truth labels for collaterals were the consensus of three neuroradiologists in their study. Following a similar strategy to our work, the authors dichotomized the collateral scores as poor and good. The authors provided no information regarding the patient demographics data and algorithm of the software. The author found that the software agreed with the consensus of the radiologists over 90%, yet they did not involve the detection of LVO on CT angiography.

Mc Louth et al. investigated the performance of a commercially available (Avicenna.ai, La Ciotat, France) deep learning product in identifying LVO^17^. The authors investigated the performance of the software on a relatively large representative US population sample consisting of CTA scans obtained with the scanners of various manufacturers. Similar to the DL model developed in this work, the software provided excellent sensitivity (98.1%) and specificity (98.2%) in identifying LVO on CTA in their study. However, they did not provide in-depth information about the inner workings of the software, and the software was unable to assess collateral blood flow.

Amukotuva et al. investigated the commercially available software (RAPID CTA, RAPID 4.9, iSchemaView, Menlo Park, CA) in their single-center large sample^14^. The software version used in their study relied on traditional image processing methods. The authors found that the software had relatively good sensitivity (92%) yet low specificity (81%) using the consensus of two neuroradiologists as the ground truth. The authors suggested that the software produced many false positives due to vascular asymmetry and cautioned against its use as an alternative to a radiologist. We believe that the low performance of their software compared with the DL software mentioned above and the nnDetection model is another good example, depicting the superiority of DL methods over traditional approaches.

Yahav-Dovrat et al. retrospectively used a commercially available DL software (Viz LVO (Viz.ai) in their large-scale sample from a comprehensive stroke center^31^. The software provided a sensitivity of 81% in identifying LVO. However, the authors did not assess the collateral status on CTA. The software used in their work mainly relied on a segmentation model. The software automatically segmented ICA-T, MCA-M1, and MCA-M2 on both sides, compared the length of the segmentations, and triggered an LVO alert if one side was shorter compared with the other. We speculate that approaching the task of LVO detection on CTA as a 3D-object detection problem is much akin to that of radiologists who tend to visually assess ICA-T, MCA-M1, and MCA-M2 on both sides for any area with contrast filling defects, potentially explaining the higher performance of our model. Furthermore, 3D object detection models can be trained with bounding boxes, which are much easier to provide than segmentation masks.

Stib et al.^16^ designed their in-house model on single-phase and multiphase CT angiography images in assessing LVO. They first projected the raw images into a single 3D-MIP image and then assessed the presence of LVO and graded collaterals using a classification DL model. Notably, the model trained on single-phase CTA scans provided a low performance with a sensitivity of 77%, while the model trained on multiphase images reached a sensitivity of 100%. On the other hand, the specificity of the latter model still remained low (77%). Their model could not provide location or segmentation information for LVO, and they did not test its performance on an external data. Furthermore, the authors did not investigate the yields of their model in assessing collateral status. We suppose that not providing location information during the training (i.e., 3D boxes) might deteriorate the models' performance in their study.

Wolf et al.^18^ used the commercially available DL software (StrokeViewer, Nicolab B.V.) to assess collateral scoring in 1024 patients derived from the Multicenter Randomized Clinical Trial of Endovascular Treatment of Acute Stroke Registry. The authors found that the dichotomized collateral scores provided by the software had a good agreement with eight experienced radiologists. Unfortunately, Wolf et al. did not provide information about the DL methodology and did not assess LVO detection performance^18^.

Several limitations to this present study should be acknowledged. First and foremost, although the entire study sample was considerably large, the external test data was relatively small. We chose to spare a considerable chunk of the data to train the DL model due to the fact that DL models often provide more robust and generalizable performance when being fed by large-scale data. Second, we trained the collateral scoring system using semi-quantitative scores assigned by human radiologists, undeniably transmitting their biases to the model and hindering the quality of the "ground truth". One possible solution to this might be implementing fully quantitative collateral scoring and formulating the task of collateral scoring as a regression problem to train DL models^15^. Nevertheless, radiologists who assigned the initial scores had experience in stroke imaging and provided moderate inter-rater agreement. Furthermore, we tried to ensure the quality of the final collateral scores by resolving inconsistencies in a joint meeting attended by an expert neuroradiologist.

Third, our DL model was trained on single-phase CTA scans as it was the de-facto method used to obtain CTA in our sample. Although some earlier studies documented that single-phase CTA can be a reliable predictor of clinical and radiological outcomes^7^, others showed that multiphase CTA offered better outcome prediction than single-phase CTA^32,33^. These papers documented that single-phase CTA underestimated the collateral status compared with multiphase CTA. Similarly, Stib et al. pointed out the higher performance of DL models on multiphase data for identifying LVO^16^. Nevertheless, our approach can easily apply to multiphase CTA scans, possibly improving its collateral scoring performance. Fourth, our study was unable to directly compare the performance of our model with existing commercial software due to limited access during the research period. Nevertheless, we recognize the importance of such comparisons and are committed to conducting in-depth, head-to-head evaluations with various commercial solutions in future studies. These comparative analyses will preferably involve more extensive multi-center test data, allowing for a robust assessment of the model's generalizability, reliability, and effectiveness in diverse settings and populations.

In conclusion, a self-configuring 3D nnDetection model can accurately identify LVO on single-phase CTA scans, providing the occlusion location with understandable bounding boxes. Further, using a multi-tasking approach, the same model can accurately provide semi-quantitative collateral scores, providing a one-stop-shop approach for automated stroke diagnostics on CTA in patients with LVO.

## Data Availability

The datasets generated during and/or analyzed during the current study are available from the corresponding author upon reasonable request.
